# Tissue-specific features of innate lymphoid cells in antiviral defense

**DOI:** 10.1038/s41423-024-01161-x

**Published:** 2024-04-29

**Authors:** Sytse J. Piersma

**Affiliations:** 1 Division of Rheumatology, Department of Medicine, Washington University School of Medicine, St. Louis, MO 63110 USA; 2 Siteman Cancer Center, Washington University School of Medicine, St. Louis, MO 63110 USA

**Keywords:** NK cell, innate lymphoid cell, tissue microenvironment, virus infection, anti-viral immunity, Innate lymphoid cells, Infection, Viral infection

## Abstract

Innate lymphocytes (ILCs) rapidly respond to and protect against invading pathogens and cancer. ILCs include natural killer (NK) cells, ILC1s, ILC2s, ILC3s, and lymphoid tissue inducer (LTi) cells and include type I, type II, and type III immune cells. While NK cells have been well recognized for their role in antiviral immunity, other ILC subtypes are emerging as players in antiviral defense. Each ILC subset has specialized functions that uniquely impact the antiviral immunity and health of the host depending on the tissue microenvironment. This review focuses on the specialized functions of each ILC subtype and their roles in antiviral immune responses across tissues. Several viruses within infection-prone tissues will be highlighted to provide an overview of the extent of the ILC immunity within tissues and emphasize common versus virus-specific responses.

## Introduction

Innate lymphoid cells (ILCs) rapidly respond to invading pathogens and cancer and can be categorized into five subsets, including natural killer (NK) cells, ILC1s, ILC2s, ILC3s, and lymphoid tissue inducer (LTi) cells, which develop from common innate lymphoid progenitors [1]. These subsets have functional parallels with CD8+ T cells and CD4+ T helper subsets [2]. Both NK cells and ILC1s produce type I cytokines, including IFNγ and TNFα, except for murine NK cells, which can produce IFNγ but not TNFα under steady-state conditions. NK cells express a wider repertoire of activation and inhibitory receptors, such as Ly49 in mice and KIR in humans, and are more cytotoxic than ILC1s [1, 3, 4]. NK cells and ILC1s employ the transcription factor T-bet for maturation and development, respectively [3, 5, 6]. NK cells require the transcription factor eomesodermin (Eomes), while ILC1s develop independently of Eomes [5–7]. In contrast, ILC1s require the transcription factor Hobit (encoded by *ZNF683*) for their development, while NK cell numbers are not affected by Hobit expression [8–10]. ILC2s are characterized by their ability to produce type II cytokines, including IL-4, IL-5, and IL-13, and their requirement for the transcription factor GATA-3 [1, 11–15]. ILC3s can produce type III cytokines, including IL-22 and IL-17, and utilize the transcription factor RORγt in mice, and IL-17A-producing ILC3s require RORC in humans [16–21]. Initially, LTi cells were grouped within ILC3s, as LTi cells also require RORγt and produce IL-22 and IL-17 [22]. LTi cells have since been classified as their own subset [1]. In contrast to ILC3s, LTi cells do not require the transcription factor PLZF but express neuropilin-1 and are required for the formation of secondary lymphoid organs, including lymph nodes and Peyer’s patches [23–28]. Phenotypic markers of different subsets of murine and human ILCs have been extensively reviewed elsewhere [1, 29, 30].

Despite the unique transcription factor requirements for each ILC subtype, plasticity between subtypes has been observed, especially in response to pathogen infection and cancer (excellently reviewed elsewhere [31–35]). For example, NK cells can acquire an ILC1-like phenotype, including downregulation of Eomes, in response to cancer and *Toxoplasma gondii* infection [36, 37]. Moreover, ILC3s can convert into ILC1-like cells that produce IFNγ in vitro [38]. Within the human tonsil, there is a spectrum of ILC1s and ILC3s, including an intermediate population that features both ILC1s and ILC3s [39]. RNA velocity analysis indicated that some of these cells were converted from ILC3s to ILC1s. Indeed, the transfer of ILC3s into humanized mice results in their acquisition of more ILC1 features in the spleen than in the liver, indicating tissue specificity for this process [39]. Thus, different ILC subtypes can acquire the traits of other subtypes [29], indicating that ILCs can adapt to specific needs within the tissue microenvironment. However, ILC plasticity makes it more difficult to attribute functions to specific ILC subsets and may cause misinterpretation of the role of different ILC subsets in antiviral immunity.

Early characterization of ILC subsets was based on surface markers, transcription factors, and cytokine production [1]. As the markers used to characterize these subsets differ between some studies and are subject to plasticity, it is difficult to identify specific subsets in some cases. Characterization of tissue-specific ILCs by single-cell RNA sequencing (scRNA-seq) has greatly improved the characterization of similarities and differences within and between ILC subsets in an unsupervised manner [39–46]. Together, these studies further enhance our understanding of the different ILC subtypes as well as their role in tissue-specific antiviral immunity, which will be discussed below.

## NK cells in antiviral defense

**Fig. 1:**
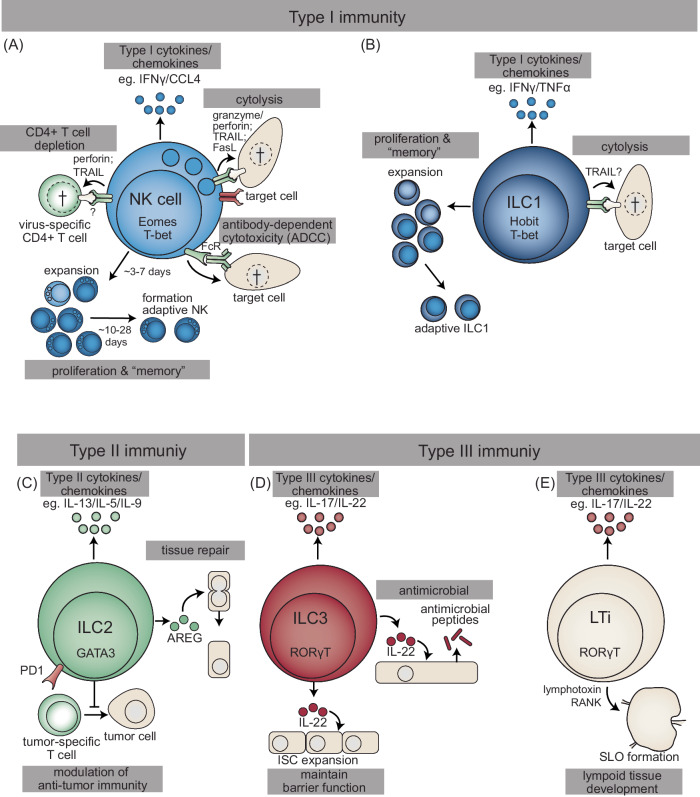
Innate lymphoid cell (ILC) effector functions. **A** NK cells are characterized by the expression of the transcription factors eomesodermin (Eomes) and T-bet (encoded by TBX21). Activated NK cells produce type I cytokines, including the signature cytokine IFNγ. NK cells recognize target cells via a combination of activation and inhibitory receptors and subsequently kill the target cells in a perforin/granzyme-, FAS-, or TRAIL-dependent manner. NK cells also recognize and kill antibody-coated target cells through Fc receptors (FcRs), resulting in antibody-mediated cytotoxicity (ADCC). NK cells have the capacity to kill activated virus-specific CD4+ T cells via TRAIL- and perforin-dependent mechanisms, but their receptor‒ligand interactions have not yet been elucidated. In response to activation, NK cells expand and peak around Day 7, after which the population contracts to form a long-lived adaptive compartment. **B** ILC1s are characterized by the expression of the transcription factors Hobit (encoded by ZNF683) and T-bet. ILC1s produce type I cytokines, including IFNγ and TNFα. ILC1s expand, contract, and form an adaptive compartment. Based on depletion studies, ILC1s have been implicated in directly recognizing and killing virus-infected cells in a TRAIL-dependent manner. **C** ILC2s are characterized by high expression of the transcription factor GATA3 and produce type II cytokines. ILC2s can produce amphiregulin (AREG), which is involved in tissue repair. Within the tumor microenvironment, ILC2s can express the checkpoint programmed cell death 1 (PD1) and modulate tumor-specific T-cell responses. **D** ILC3s express the transcription factor RAR-related orphan receptor γT (RORγT) and produce type III cytokines, including IL-17 and IL-22. IL-22 produced by ILC3s stimulates the production of antimicrobial peptides and the expansion of intestinal stem cells (ISCs) to maintain barrier function. **E** Lymphoid tissue inducer (LTi) cells are also characterized by the expression of the transcription factor RORγT and the production of type III cytokines. LTi induces the formation of secondary lymphoid organs (SLOs) in a manner dependent on lymphotoxin and receptor activator of nuclear factor kappa B (RANK)

Natural killer cells were the first identified ILC subtype and were named based on their “natural” cytotoxicity against lymphoma cells, including the prototypic YAC-I target cell line [47, 48]. NK cells are instructed through surface receptors and cytokines that result in the initiation of various effector functions (Fig. 1A). NK cells express an array of activating and inhibitory surface receptors, and the resulting balance between activating and inhibitory signals determines whether the NK cell is activated. This has been well exemplified in NK cell recognition of major histocompatibility complex (MHC)-I-deficient cells termed “missing self” [49, 50]. This is particularly relevant for virus infections, as many viruses avoid CD8 + T-cell recognition by manipulating MHC-I surface expression. As a result, these virus-infected cells become potential “missing self” targets and are sensitive to NK cell-mediated lysis.

The inhibitory Ly49 receptors in mice and killer cell immunoglobulin-like receptors (KIRs) in humans recognize MHC-I on healthy cells, thereby preventing NK cell activation. NK cells are educated to recognize self-MHC-I through Ly49 and KIR receptors, and these NK cells exhibit increased responsiveness through a process termed licensing [51, 52]. NK cells also express Fc receptors that can mediate lysis of antibody-coated virus-infected cells through a process called antibody-dependent cellular cytotoxicity (ADCC), which has been associated with the control of influenza and HIV infection [53, 54]. Upon recognition of target cells, NK cells can directly recognize and lyse virus-infected cells and produce proinflammatory cytokines, including their signature cytokine IFNγ (reviewed in [55]).

In addition to recognizing infected cells, NK cells can also directly affect adaptive immunity during virus infection. For example, NK cells have been reported to kill virus-specific CD4 + T cells in a perforin- and TRAIL-dependent manner in response to lymphocytic choriomeningitis virus (LCMV) and murine cytomegalovirus (MCMV), respectively [56, 57]. However, NK cells play a critical role in the control of virus infection, for which there are independent lines of evidence that will be discussed below.

Studies in animal models have shown a critical role for NK cells in controlling various viral infections. Early studies in mice revealed that NK cells can protect against virus infections, including lymphocytic choriomeningitis virus (LCMV) and murine cytomegalovirus (MCMV) [58]. MCMV has emerged as a prototypic virus for studying antiviral NK cell immunity and has revealed many facets of NK cell biology. MCMV-specific NK cell immunity has been best characterized in C57BL/6 mice and is dependent on recognition of the virus-encoded MHC-I ortholog m157 by the activation receptor Ly49H [59–63]. This interaction is sufficient to mediate protection against otherwise lethal infection [60].

In addition to the dominant Ly49H-m157 interaction, other NK cell recognition mechanisms against MCMV have been described, including missing self recognition and other activation receptors [64–66]. Control of MCMV depends on the direct killing of MCMV-infected targets and the production of the proinflammatory cytokine IFNγ [67–70]. In response to MCMV infection in C57BL/6 mice, Ly49H-specific NK cells expand, contract, and form a long-lived compartment analogous to that of memory T cells [71, 72]. These MCMV-induced adaptive Ly49H+ NK cells include 2 distinct populations, one that is transcriptionally equivalent to conventional NK cells and one that is more transcriptionally similar to ILC1s, indicating that adaptive NK cells encompass features from both NK cells and ILC1s [73].

Evidence is emerging that these adaptive NK cells are also present in humans [74]. Analogous to MCMV and Ly49H expression on NK cells, human cytomegalovirus (HCMV) induces an adaptive NKG2C + NK cell population in seropositive donors that is characterized by decreased expression of PLZF, FcRγ, EAT-2, and Syk [75–78]. Thus, NK cells can protect against lethal virus infection in animal models and can form an adaptive compartment that has also been identified in humans.

Second, combinations of specific KIR receptors and their HLA ligands have been associated with improved viral control [79]. The combination of activated KIR3DS1 and HLA-Bw4-80I is associated with increased control of HIV infection [80, 81]. Furthermore, HLA-Bw4-80I+ patients display increased expansion of KIR3DS1 + NK cells during acute HIV infection, and KIR3DS1 + NK cells respond to infected HLA-Bw4-80I + CD4 + T cells [82, 83]. Control of HIV infection in humanized mice has been reported to be dependent on NK cells [84]; however, the specific KIR-HLA interactions were not addressed in this study. Thus, population-based genetic studies have revealed more evidence clarifying the importance of NK cells in controlling virus infection, which is supported by experimental models.

Third, patients with specific NK cell defects clinically present with recurrent virus infections. The first evidence that NK cells are critical in human antiviral defense came from a case report of an adolescent patient who presented with a severe herpesvirus infection and lacked natural killer cells [85]. To date, more than 50 individuals with congenital NK cell deficiency (NKD) have been identified [86]. These patients frequently suffer from recurrent viral infections, including herpes and papillomavirus infections, and they can lead to fatal outcomes [86]. These congenital errors can be divided into patients with classical NKD, which is characterized by low NK cell numbers, and patients with functional NKD, who have normal NK cell numbers but decreased NK cell cytolytic function. Classical NKDs are caused by deficiencies in transcription factors, such as *IRF8* and *GATA2* [87, 88], as well as defects in genes associated with the cell cycle, including *MCM4*, *MCM10*, *GINS1*, and *RTEL1* [89–92]. Functional NKDs have been identified in patients with defects in the surface receptor *FCGR3A* and the signaling molecule *PLCG2* [93, 94]. Taken together, these human syndromes highlight the role of NK cells in viral control, particularly in recurrent infections.

Finally, several viruses encode proteins that specifically interfere with NK cell function and avoid being cleared by NK cell immunity (reviewed in [55, 95–97]). In particular, viruses with large genomes, such as herpesviruses and poxviruses, encode numerous dedicated immune evasion molecules. A good example of a viral immune evasion molecule that targets NK cells is the viral MHC-I ortholog encoded by various herpesviruses: HCMV encodes the MHC-I ortholog UL18 [98, 99], MCMV encodes the MHC-I ortholog m157 [62, 63, 100], and rodent herpesvirus Peru encodes the nonclassical MHC-I ortholog pQa-1 [101]. Thus, these viral immune evasion molecules suggest that there is evolutionary pressure on the respective viruses to develop strategies to counteract NK cell immunity.

Taken together, these lines of evidence in human and animal models underline the importance of NK cells in antiviral immunity.

### Tissue-specific features of NK cells

NK cells in humans can be identified based on their expression of CD56 and NKp46 and lack of CD3 expression. Human NK cells can be further subdivided into 2 separate populations, namely, CD16+CD56^dim^ and CD16-CD56^bright^ populations. The CD56^dim^ population is characterized by its high cytolytic potential, while the CD56^bright^ population has the capacity to produce greater levels of cytokines and chemokines [102, 103]. In contrast, mouse NK cells in C57BL/6 mice express NKp46, NK1.1, and CD49b but lack CD3 expression. Mouse NK cells can be further subdivided based on CD11b and CD27 expression [104, 105]. CD27 + CD11b- cells are in the immature stage and mature through the CD27 + CD11b+ phenotype to become the most mature CD27-CD11b+ phenotype, which is characterized by the greatest potential for effector function. Despite these differences in the identification of NK cells in humans and mice, scRNA-seq of NK cells isolated from peripheral blood and the spleen revealed that human and mouse NK cells are transcriptionally similar and can be grouped into 2 major subsets [43]. This study revealed that human CD16-CD56^bright^ NK cells have a transcriptional profile similar to that of mouse CD27 + CD11b- NK cells, while human CD16+CD56^dim^ NK cells have a transcriptional signature similar to that of mouse CD27-CD11b+ NK cells. Thus, despite differences in the identification of NK cells in humans and mice, NK cells are transcriptionally similar in these species, suggesting that NK cell biology is largely preserved across species.

NK cells are relatively abundant in blood, infiltrate different tissues, and are capable of recirculating between tissues. Their ability to recirculate is well exemplified in parabiosis experiments where NK cells from one parabiont seed to organs from the other congenic parabiont, while ILC1, ILC2, ILC3, and LTi cells do not recirculate [3, 106, 107]. Despite their ability to circulate, NK cells acquire tissue-specific traits in several organs [25, 42, 108–110]. CD16+CD56^dim^ NK cells predominate in blood-rich organs, including the bone marrow, spleen, and lungs, while CD16-CD56^bright^ NK cells predominate in lymphoid and mucosal tissues [109]. In human mucosal tissues, NK cells can acquire markers associated with ILC1s, including increased CD103 and CD49a expression and decreased expression of the cytolytic proteins perforin and granzyme B, potentially as a result of TGFβ signaling [108, 110]. In mouse mucosal tissues such as the small intestine lamina propria (siLP), NK cells acquire tissue-specific transcriptional signatures that are also found in other siLP ILC populations [25]. These transcriptional changes may cause fewer NK cells to recirculate, which has been found in parabiosis experiments [107]. NK cells in the mouse salivary gland and uterus also acquire tissue-specific transcriptional profiles and are predominantly tissue-resident cells [3, 42, 107]. TGFβ in the tissue microenvironment signals through TGFBR2 on NK cells to acquire ILC1-associated markers, including CD49a, CD103, and CD69 [42, 111]. However, the absence of TGFβ signaling did not impact the tissue residency of these cells [111]. Thus, NK cells acquire tissue-specific features that can impact function and tissue residency.

## ILC1

ILC1s were first identified as organ-specific NK cells in several organs, including the thymus [112], liver [104], pancreas [113], and intestine [114]. Using parabiosis experiments, these cells were found to be tissue-resident and were referred to as tissue-resident NK cells in some cases [3, 106, 107]. However, subsequent studies have shown that ILC1s develop independently of NK cells and comprise a distinct lineage [1, 2, 6, 7]. ILC1s are characterized by the potent production of type I cytokines, including IFNγ and TNFα (Fig. 1B). ILC1s and NK cells have largely overlapping receptor repertoires and can produce type I cytokines; as a result, these cells can be difficult to separate experimentally [1]. Moreover, during certain conditions such as inflammation, this repertoire can change, and receptors that can be used to discriminate in the steady state are then expressed by both populations. For example, NK cells can acquire the ILC1 marker CD49a in response to MCMV infection [115]. Currently, the expression of Eomes on NK cells versus Hobit expression on ILC1s is the most reliable way to differentiate between these populations. However, a study using Hobit-reporter mice showed that NK cells may express low levels of Hobit in response to cytokine stimulation and MCMV infection [10]. Moreover, low numbers of ILC1s can be detected in Hobit-deficient mice [9, 10], suggesting that a minor population of ILC1s can develop in a Hobit-independent manner.

The signature cytokines IFNγ and TNFα produced by ILC1s are known for their direct antiviral and immunomodulatory effects, suggesting that they may play key roles in antiviral defense. Indeed, ILC1s in the liver mediate IFNγ-dependent antiviral effects against MCMV in the earliest stages of infection [116]. While ILC1s were initially described as type I cytokine-producing cells, later studies showed that ILC1-like cells within the tumor microenvironment also have cytolytic potential [117, 118]. These observations highlight clear parallels between ILC1s and CD4 + T cells, which also have the potential to acquire cytotoxic functions in the cancer setting and during viral infections [119]. There are also indications that ILC1s mediate cytolytic functions during virus infection. MCMV encodes ORF m166, which blocks TNF-related apoptosis-inducing ligand (TRAIL)-mediated lysis [120]. In response to MCMV, TRAIL is specifically expressed by ILC1s but not conventional NK cells, and m166-deficient MCMV shows increased control in the liver and salivary glands, suggesting that ILC1s can control m166-deficient MCMV in a TRAIL-dependent manner [121]. Moreover, these studies also suggest that MCMV has developed ILC1-specific immune evasion strategies. Studies directly investigating the role of ILC1s in TRAIL-dependent viral control are required to further validate these findings. Thus, ILC1s have been linked to antiviral immunity; however, further characterization is required to understand the role of ILC1s in human antiviral immunity and may reveal additional virus-specific features of ILC1s.

### Tissue-specific features of ILC1s

Human ILC1s can be divided into two subsets, namely, ILC1s, which produce IFNγ and lack cytolytic potential, and a second subset termed ieILC1s, which are characterized by TGFβ imprinting and CD103 expression [29]. IeILC1s were first identified in the gut epithelium and tonsils and are transcriptionally similar to CD4+ and CD8+ tissue-resident T cells [29, 114]. Subsequent studies using scRNA-seq and other high-dimensional techniques revealed that ILC1s can cross many human tissues, including the liver, spleen, lung, tonsil, and adipose tissue [41, 108–110, 122]. Within tissues, ILC1s maintain their core signature but express tissue-specific transcripts, as was first indicated in the mouse spleen, liver, lamina propria, and intraepithelium in the small intestine [25]. A comparison of different human tissues revealed differences between spleen and jejunum ILC1s, with spleen ILC1s displaying increased functional potential, as evidenced by increased transcripts of cytokines and chemokines [108]. Moreover, a recent study combining scRNA-seq data from mouse ILC1s with a meta-analysis of available human scRNA-seq datasets revealed tissue-specific and interspecies ILC1 gene signatures [44]. In summary, ILC1s are present across a range of tissues but acquire tissue-specific features dependent on the microenvironment.

## ILC2

ILC2s are tissue-resident and are characterized by their capacity to produce the type II cytokines IL-4, IL-5, and IL-13 and they can also produce the inhibitory cytokine IL-10 [11, 13, 107, 123–125]. These cells are instructed by cytokines, including IL-25, TSLP, and IL-33 (reviewed in [31]). ILC2s are key in the innate immune response to parasites, including *Nippostrongylus brasiliensis* [107, 126]. After infections are cleared, ILC2s contribute to tissue repair by producing amphiregulin (AREG) [127, 128]. ILC2s can be identified by high expression of GATA-3, the IL-33 receptor chain ST2 in most tissues, and the prostaglandin D2 receptor CRTH2 in humans [14, 129, 130]. ILC2s display specific phenotypes within the tissue in which they reside, including immature, activated, and IL-10-producing suppressive phenotypes [31]. Although ILC2s have not been found to participate directly in antiviral immunity, ILC2s have been reported to impact antiviral immunity in different manners. ILC2s in the lungs of COPD patients have been shown to convert to ILC1s, resulting in IFNγ and augmented antiviral inflammation [131]. Moreover, ILC2s have been shown to impact the adaptive immune response in cancer. ILC2s infiltrating pancreatic ductal adenocarcinomas express the inhibitory checkpoint PD-1, and anti-PD-1 immunotherapy augmented by IL-33 administration results in increased antitumor immune responses [132]. Thus, ILC2s impact the immune response in multiple ways (Fig. 1C). The role that ILC2s play in the context of virus infection will be discussed below. As ILC2s have been relatively recently identified, our understanding of ILC2 function in virus infection is likely incomplete.

### Tissue-specific features of ILC2s

While ILC2s can be detected in most tissues analyzed, under steady-state conditions, they seem to be relatively rare in nonmucosal tissues, being more prominent in mucosal tissues and highly prevalent in skin [45, 108, 110]. Moreover, their numbers can increase under pathological conditions [31, 110]. In the intestines, ILC2s can be activated by IL-25 and IL7, while the combination of IL-7 and IL-33 is the main stimulus in other tissues [31]. Moreover, intestinal ILC2s can migrate in response to helminths to distant tissues [133]. Thus, ILC2s are prominent at barrier sites and have the capacity to migrate to other tissues under inflammatory conditions.

## ILC3s and LTi

ILC3s were identified as tissue-specific NK cell subsets, including NK-22 cells, in early studies because they express multiple NK cell receptors, including NKp46 and NKp44, and have the capacity to produce IL-22 and IL-17 [16, 18, 19, 21]. ILC3s are abundant in mucosal tissues, including the tonsils and intestine. ILC3s produce IL-22 under homeostatic conditions, which is important for maintaining intestinal homeostasis and barrier integrity (Fig. 1D) [134]. IL-22 was shown to be particularly important for early host defense against *Citrobacter rodentium* [135], suggesting that ILC3s may contribute to the control of this pathogen. Indeed, ILC3s produce increased amounts of IL-22 in response to *C. rodentium* infection, which contributes to decreased susceptibility [19, 21, 136]. IL-22 produced by ILC3s induces antimicrobial peptides and stabilizes the epithelial barrier [16, 135, 137]. ILC3s respond to IL-23 to produce IL-17, IL-22, and IFNγ [138]. Moreover, ILC3s can limit adaptive Th17 responses through direct antigen presentation by MHC-II on ILC3s [139]. Although experimental data showing that ILC3s directly recognize virus-infected cells, such as NK cells and ILC1s, are lacking, ILC3s can indirectly impact viral immunity, which will be discussed below.

Unlike other ILCs, LTi cells are instrumental in the development of secondary lymphoid organs in the fetus in a lymphotoxin- and RANK-dependent manner (Fig. 1E) [23, 140]. Tertiary lymphoid structures are formed during inflammation, such as virus infection and oncogenesis; however, this lymphoid tissue development is largely independent of LTi cells [141], indicating that infection-induced lymphoid structures can develop in the absence of LTi cells. LTi cells are difficult to separate from ILC3s because they share many characteristics, including cell surface markers and transcription factor dependence, including RORγT [1]. However, these cells express neuropilin-1 but not NKp46 or NKp44 [25, 26]. Taken together, LTis are unlikely to directly participate in antiviral immunity; however, they are required for a functional adaptive antiviral immune response, as they are essential for secondary lymphoid tissue development.

### Tissue-specific features of ILC3s

ILC3s within tissues express distinct surface markers dependent on the microenvironment [30]. Within different tissues, murine ILC3s express distinct chemokine receptor profiles: NKp46+ lamina propria ILC3s express CXCR6, mesenteric LN ILC3s express CCR7, intestinal ILC3s express integrin α4β7 and CCR9, and skin ILC3s express CCR6 [142–144]. Tissue-specific phenotypes and functions of human ILC3s have been reported [30]. Human tonsils and other mucosal tissues contain 2 specialized ILC3 subsets that are not found in nonmucosal tissues [41, 110, 145]. These include a subset with increased production of IL22 and another with increased expression of CD69, CD25, and ICOS [110, 145]. Thus, ILC3s have several tissue-specific features that likely reflect their role within the tissue and potentially affect antiviral immunity, which will be discussed below.

## Tissue-specific antiviral ILC immunity

As described above, NK cells are better characterized for their role in antiviral immunity compared to helper ILC subsets. However, recent studies have revealed that helper ILC subsets participate in antiviral immunity, especially within tissues. The next section will highlight the involvement of the different ILC subsets in antiviral immunity across different tissues. Within specific tissues, several viruses will be highlighted to indicate how the ILC immunity contributes to or perturbs antiviral immunity.

## Respiratory infections

Infections of the respiratory tract, including the SARS-CoV-2, influenza virus and respiratory syncytial virus (RSV), are among the main modes of transmission of these viruses. ILCs have been shown to promote antiviral immunity and cause pathology in response to viral infection. These features of ILCs in respiratory infections will be discussed below.

### Influenza infection

**Fig. 2:**
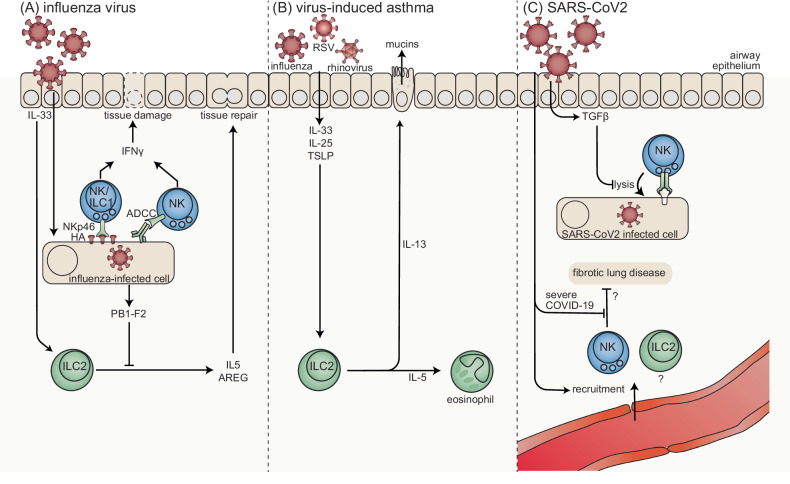
ILCs in viral pulmonary infections.**A** NKp46+ ILCs can protect against lethal intranasal influenza infection. Influenza HA can be recognized by NKp46 and induce NK cell-mediated lysis. In immunized settings, NK cells contribute to the control of influenza infection through ADCC. The IFNγ produced in response to influenza infection causes tissue damage, which is counteracted (in part) by ILC2s. ILC2s mediate tissue repair by producing AREG and IL-5 in response to influenza infection, which is increased by IL-33 administration. The influenza virus encodes PB1-F2, which acts as an immune evasion molecule by repressing ILC2 IL-5 responses. **B** Respiratory syncytial virus (RSV), influenza, and rotavirus have been implicated in virus-induced asthma. In response to infection, IL-33, IL-25, and thymic stromal lymphopoietin (TSLP) stimulate ILC2s to produce IL-5 and IL-13, which stimulate the recruitment of eosinophils and increase mucus production, respectively. **C** Severe acute respiratory syndrome coronavirus 2 (SARS-CoV-2)-infected cells can be lysed by NK cells via a combination of activating receptors. The cytolytic capacity of NK cells is inhibited by transforming growth factor β (TGFβ) during the first weeks of infection. SARS-CoV-2 infection leads to the recruitment of NK cells and possibly ILC2s from the circulation into the lungs. Severe coronavirus disease (COVID-19) is associated with decreased antifibrotic activity by NK cells, indicating a potential role in fibrotic lung disease

Influenza is a prototypic respiratory infection that causes substantial mortality, particularly in older individuals [146]. While the immune system is critical for controlling influenza infection, influenza also triggers excessive inflammation that can cause fatal disease in some cases [147]. Recent studies have implicated ILCs in these processes (Fig. 2A). ILCs have been implicated in protection against lethal intranasal (i.n.) influenza infection in an NKp46-dependent manner [148], likely through the recognition of influenza HA on infected cells [149]. In addition to direct recognition, NK cells have also been implicated in ADCC-mediated control of influenza infection in macaques within the bronchioalveolar space [150]. Murine NK cells are recruited to the lungs in response to influenza in a CXCR3- and CCR5-dependent manner [151]. Human NK cells, including CXCR3 + NK cells, accumulate in the lungs of individuals with acute influenza infection and exhibit a hyperresponsive phenotype [152]. Thus, NK cells have the capacity to recognize influenza-infected cells within infected tissues and contribute to viral control, but additional studies are needed to further define the role of NK cells in antiviral control.

Airway ILCs have been reported to protect against influenza-induced lung damage (Fig. 2A), including epithelial integrity and airway remodeling, in response to influenza infection, which is mediated by IL-33 and AREG [127]. A subset of BATF+ ILC2s mediate wound healing, and BATF deficiency leads to the acquisition of pathogenic ILC3-like functions [153]. GITR expression by lung ILC1s can inhibit IFNγ production in response to influenza infection in mice [154]. Moreover, mice deficient in IFNγ show decreased immunopathology in response to influenza infection, which is associated with increased IL-5 production by ILC2s, suggesting that ILC2s protect against immunopathology [155]. ILC2s can also convert to IFNγ-producing ILC1s in response to influenza, which potentially contributes to COPD [131]. Intranasal administration of IL-33 promoted vaccine-induced protection in an influenza mouse model, which was mediated by ILC2s [156]. Influenza encodes PB1-F2, a protein that represses ILC2 IL-5 responses, leading to decreased tissue integrity and survival [157], suggesting that influenza has evolved immune evasion mechanisms that target ILC2s. Taken together, these studies indicate that lung-resident ILC1s and ILC2s play a key role in influenza-induced immunopathology.

### Virus-induced asthma

In both human and mouse studies, ILC2s have been hypothesized to be involved in virus-induced asthma pathology (Fig. 2B). Murine lung-resident ILC2s produce IL-5 in response to influenza infection, resulting in increased eosinophil recruitment, which may cause asthma exacerbation [158]. This hypothesis is further supported by a study in asthma patients demonstrating increased baseline ILC2s in these patients, which was associated with increased type II cytokines and viral load in response to experimental rhinovirus infection, suggesting a role for ILC2s in virus-induced immunopathology in asthma patients [159, 160]. In infants with respiratory syncytial virus (RSV) infection, elevated ILC2s are associated with disease severity [161]. Moreover, neonatal rhinovirus, RSV, and influenza infection can induce ILC2 expansion in young mice, suggesting that early viral infection may contribute to asthma development [162–164]. Neonatal virus-induced ILC2 expansion involves IL-25, IL-33, and TSLP and can be blocked by IFNγ or IL-1β administration [162, 163, 165–167]. Moreover, ILC2s induce mucin production in response to RSV infection [165]. Adoptive transfer studies have shown that ILC2s mediate eosinophil accumulation and immunopathology in response to RSV infection [168]. Male mice are more susceptible to ILC2- and TSLP-dependent RSV-induced allergic disease, indicating that there may be sex-specific effects on ILC2s and asthma [169]. RSV infection also induces STAT1-dependent IFNγ production by NK cells, while STAT1-deficient mice exhibit increased IL-5+ and IL-13 + ILC2s and IL-17A + ILC3s in the lung in response to RSV infection [170], suggesting that ILCs play a role in RSV infection beyond impacting asthma pathology. Collectively, these studies implicate lung-resident ILC2s in virus-induced asthma.

### SARS-CoV-2 infection

SARS-CoV-2 is the causative agent for COVID-19, which started in late 2019 [171]. NK cells reportedly participate in the immune response to SARS-CoV-2 (reviewed in [172]). SARS-CoV-2 infection induces an activated phenotype in peripheral NK cells, as indicated by the upregulation of CD69, perforin, and granzyme B, but a decreased capacity for degranulation and cytolysis [173–176]. NK cells are recruited from the periphery to infected lungs in response to SARS-CoV-2 infection (Fig. 2C), leading to decreased numbers in the peripheral blood and increased numbers in the bronchoalveolar fluid [173, 175, 177–179]. Similarly, the frequency of ILC2s was decreased in the peripheral blood of patients with severe COVID-19 compared to patients with mild disease [180, 181]. However, the role of ILC2s in disease progression is unclear. NK cells can directly recognize SARS-CoV-2-infected cells via a combination of activation receptors [176], but specific receptor-ligand interactions have not been identified. However, TGFβ, which is produced in large amounts during the first 2 weeks after infection, limits the cytolytic capacity of NK cells [176]. NK cells have also been implicated in the development of fibrotic lung disease in patients with severe COVID-19 patients because of the impaired antifibrotic activity of NK cells [182]. A recent study in nonhuman primates showed that replication-competent SARS-CoV-2 can be detected in macrophages in the bronchioalveolar fluid beyond 6 months postinfection [183]. The IFNγ produced by CD8+ T cells and NK cells is inversely correlated with virus persistence, which induces increased MHC-E expression by macrophages in the lung [183], suggesting that these macrophages escape recognition by NK cells. However, the SARS-CoV-2 nonstructural protein encodes a human MHC-E-restricted peptide that is not recognized by the inhibitory receptor NKG2A and does not impair NK cell activation, suggesting that MHC-E expression by SARS-CoV-2-infected cells does not necessarily inhibit antiviral NK cell responses in humans [184]. More studies are needed to understand the NK cell response to persistent SARS-CoV-2 infections, especially in humans. Taken together, there is strong evidence that ILCs, including NK cells within the bronchoalveolar space, contribute to viral control of SARS-CoV-2 but may inadvertently contribute to the fibrotic lung disease observed in patients with severe COVID-19.

## Dengue skin infections

Dengue virus (DENV) infection is a viral mosquito-borne disease that causes a significant global disease burden [185]. An early study reported that dengue hemorrhagic fever was associated with increased NK cell numbers in the peripheral blood [186]. Moreover, a study examining skin biopsies from patients during acute DENV infection showed that NK cells can home to the site of infection during the early phases [187]. These NK cells mainly displayed a CD56^bright^ and a less mature CD56^dim^ phenotype that was characterized by activation in response to IL-18, which was detected within the blister fluid. It is, however, not clear whether NK cells directly contribute to the control of DENV infection. NK cells reportedly respond to DENV infection in vitro in an NKp44-dependent manner [188]. Thus, NK cells accumulate within the skin in response to acute DENV infection and have the potential to recognize and lyse virus-infected cells, but more research is required to clarify how NK cells function in the anti-DENV immune response.

## Hepatic infections

Hepatic virus infections can cause acute and chronic infections leading to hepatitis and cancer. Human chronic hepatitis can be caused by distinct viruses, including hepatitis B virus (HBV), a double-stranded DNA virus, and hepatitis C virus (HCV), a single-stranded RNA virus. Moreover, experimental infections with MCMV have revealed several tissue-specific ILC features within the liver. These subjects will be discussed below.

### Experimental cytomegalovirus infections

**Fig. 3:**
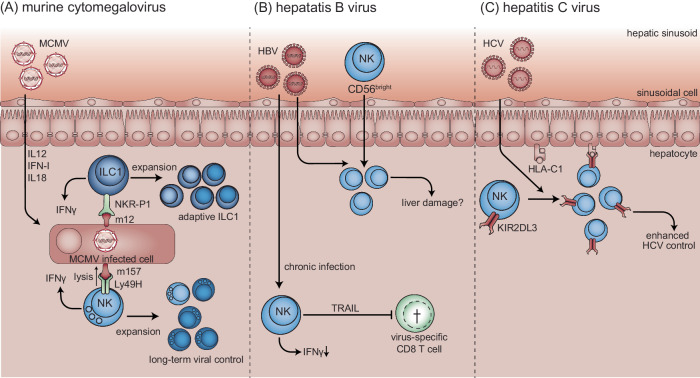
ILCs in hepatic viral infections.**A** MCMV infection induces the expression of proinflammatory cytokines, including IL-12, type I interferon (IFN-I), and IL-18, which regulate type I ILCs. ILC1s recognize MCMV-encoded m12 on infected cells through NKRP-1 receptors and restrict the viral load in an IFNγ-dependent manner. In response to MCMV infection, ILC1s expand and form adaptive compartments with increased effector functions. NK cells recognize MCMV-encoded m157 through the Ly49H activation receptor. NK cell-mediated viral control in the liver relies more on IFNγ and less on direct lysis than in the spleen. Hepatic NK cells also expand in response to MCMV infection, which is required for long-term viral control in the liver but not in the spleen. **B** Hepatitis B virus (HBV) infection results in the recruitment of CD56bright NK cells to the liver, where they potentially promote liver damage. In chronic HBV infection, NK cells display a reduced capacity to produce IFNγ but they have the capacity to restrain HBV-specific CD8 + T-cell responses in a TRAIL-dependent manner. **C** Control of hepatitis C virus (HCV) infection has been genetically associated with the inhibitory receptor KIR2DL3 and its ligand human leukocyte antigen (HLA)-C1. HCV infection induces an activated phenotype and expansion of KIR2DL3 NK cells in the peripheral blood. However, their role within the hepatic microenvironment requires elucidation

The role of different ILC subsets during acute MCMV infection in C57BL/6 mice has been thoroughly studied over the past decades. Early studies in mice showed that NK cells can protect against virus-induced hepatitis [58]. NK cells produce high amounts of IFNγ in response to MCMV infection, which has potent antiviral effects on the liver (Fig. 3A) [67]. NK cell-dependent control of MCMV infection in the liver is particularly dependent on the production of IFNγ, while m157-dependent cytolytic control is less prominent than that in the spleen [68, 70]. Moreover, STING-dependent IFN-I in the hematopoietic and stromal cell compartments contributes to the control of MCMV infection in the spleen, whereas STING plays a role only in the stromal compartment in the liver, indicating that the inflammatory environment that primes NK cells in the liver is different from that in the spleen [189]. Although NK cell expansion is not required for the control of acute MCMV infection, it contributes to long-term MCMV control in the liver in the absence of adaptive immunity [190]. This was observed mainly in the liver, while viral control in other organs, such as the spleen and salivary glands, was not affected by NK cell expansion [190]. Taken together, these studies indicate that hepatic MCMV control by NK cells requires distinct NK cell features.

ILC1s are relatively abundant within the liver, and acute MCMV infection induces early ILC1-dependent IFNγ production, even before NK cell-dependent IFNγ is detected [116]. IFNγ produced by liver ILC1s contributes to the control of hepatic MCMV infection (Fig. 3A) under conditions in which the liver is directly infected via hydrodynamic infection [116]. In response to MCMV infection, liver ILC1s expand, contract, and form an adaptive population with increased effector functions, analogous to those of NK cells [72, 191]. Memory formation requires the MCMV-encoded protein m12, which is recognized by the NKR-P1 family expressed by ILCs [191, 192]. Intriguingly, early studies describing the adaptive features of NK cells identified a CXCR6+ liver NK cell population that mediates adaptive functions against hapten sensitization and virus vaccines; this population was identified as the CD49a+ liver-resident ILC1 population in subsequent studies [106, 193]. Taken together, these studies indicate that liver-resident ILC1s contribute to viral control and can develop adaptive features.

### Hepatitis C viral infections

KIRs and their MHC-I ligands have been shown to be associated with HCV outcome [194]. This was the first report showing that homozygosity for the inhibitory receptor KIR2DL3 in combination with its ligand HLA-C1 is associated with enhanced HCV control (Fig. 3C) [195], which was confirmed in independent populations [194]. These observations are substantiated by studies of peripheral blood NK cells during HCV infection. KIR + NK cells are activated during acute HCV infection and are expanded in individuals who resolve the infection [196, 197]. Another study reported that the frequency of peripheral blood KIR2DL3 + NKG2A- NK cells is associated with protection from productive infection [198]. Taken together, these studies provide a link between HCV infection and HLA-inhibitory KIR interactions on NK cells. Characterization of the hepatic microenvironment and the effect of HCV infection on HLA expression is needed to further elucidate the role of ILCs in the antiviral immune response to HCV.

### Hepatitis B viral infections

The presence of liver-infiltrating ILCs, which have been defined as NK cells in these studies but may include ILC1s, has been studied in HBV-infected patients. TRAIL+ CD56^bright^ NK cells have been reported to increase in the periphery during flares in chronic HBV patients, which is associated with the infiltration of TRAIL+ CD56^bright^ NK cells in the inflamed liver, suggesting that these cells may promote liver damage (Fig. 3B) [199, 200]. Intrahepatic NK cells from chronic HBV-infected patients have a reduced capacity to produce IFNγ, which can be reversed by blockade of IL-10 and TGFβ [201, 202]. Moreover, NK cells can restrain HBV-specific CD8 + T-cell responses in a TRAIL-dependent manner in chronic HBV-infected patients [203]. Similarly, murine liver ILC1s restrain virus-specific CD4+ and CD8 + T-cell responses to LCMV [204]. In this viral hepatitis model, ILC2s protect against liver injury by inducing immunosuppressive neutrophils [205]. Thus, liver-infiltrating ILCs can restrict HBV-specific T-cell responses, become hyporesponsive during chronic HBV infection, and might be involved in liver damage. Additional studies are required to confirm the role of ILCs in HBV-induced liver damage.

In conclusion, within the liver microenvironment, distinct ILC populations play different roles depending on the type of virus, including mediating direct antiviral effects, affecting adaptive immunity, and causing and preventing tissue damage.

## Cardiac infections

Several viruses, including adenoviruses and enteroviruses, can cause viral myocarditis (VMC), which has been recognized as the main cause of cardiomyopathy that may lead to heart failure [206]. ILC3s have been implicated in promoting immunopathology in a mouse model of enterovirus infection using coxsackievirus 3 (CVB3)-induced VCM, which was associated with increased cardiac infiltration of ILC3s, and depletion of ILC3s resulted in decreased inflammation [207]. In response to CVB3 infection, NK cells are recruited to the myocardium in a CXCL10-dependent manner and produce IFNγ, which is associated with decreased virus titers [208]. Subsequent studies have shown that NK cells protect against the progression of inflammatory cardiomyopathy in an NKG2D-dependent manner and are restrained by myeloid-derived suppressor cells [209, 210]. Thus, type I ILCs contribute to viral control, while type III ILCs cause immunopathology in the case of CVB3-induced VCM.

## Digestive tract infections

### Poxvirus infection

**Fig. 4:**
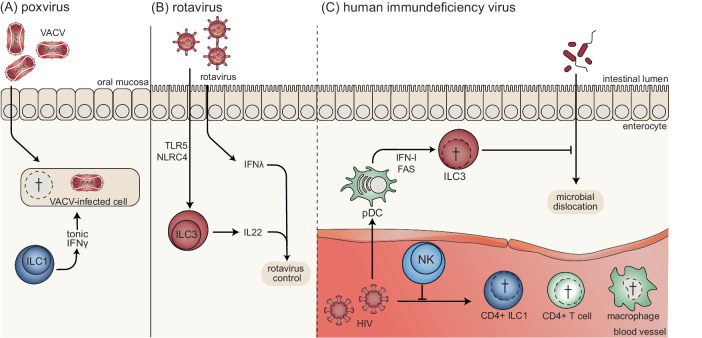
ILCs in digestive tract infections.**A** Vaccinia virus (VACV) can infect the oral mucosa and is restricted by homeostatic IFNγ production by ILC1s, which bypass poxvirus-encoded immune evasion molecules. **B** Enteric rotavirus infection leads to Toll-like receptor (TLR)5- and Nod-like receptor (NLR)C4-dependent IL-22 production by ILC3s, which mediates rotavirus control in combination with IFNλ. **C** Human immunodeficiency virus (HIV) induces plasmacytoid dendritic cells (pDCs) to restrict the number of ILC3s in an IFN-I- and FAS-dependent manner. As a result, barrier function is decreased in HIV-infected individuals, which can lead to microbial dislocation. HIV can infect CD4 + T cells, macrophages, and CD4 + ILC1s. NK cells can restrict HIV infection, which is associated with specific KIR and HLA genotypes

ILC1s patrol the oral mucosa and have been shown to restrict vaccinia virus infection in an IFNγ-dependent manner starting 9 hours postinfection (Fig. 4A) [211]. Vaccinia virus is an attenuated poxvirus that has been used to eradicate variola virus, the causative agent of smallpox [212]. Poxviruses are large DNA viruses whose genome encodes immune evasion molecules that protect infected host cells from immune attack. However, mucosal ILC1s produce IFNγ, which upregulates antiviral genes in the uninfected epithelium, providing a first-line defense against virus infection [211].

### Rotavirus infection

Rotavirus is a leading cause of childhood gastroenteritis that directly infects enterocytes [213]. Protection against rotavirus infection was identified to be independent of adaptive immunity and type I and type II IFNs [214]. This innate control of rotavirus infection requires TLR5, NLRC4, and IL-22 [214]. A subsequent study showed that IL-22 is produced by ILC3s in response to infection and synergizes with IFNλ (a type III IFN) to control rotavirus infection (Fig. 4B) [215]. Thus, when adaptive immunity is not fully developed, ILC3s can contribute to antiviral immunity in response to enteric infection, especially early in life.

### HIV infection

HIV infects immune cells, including CD4 + T cells and macrophages, causing immunodeficiency that is characterized by early and sustained loss of gut integrity [216]. Dysregulation of the intestinal barrier can lead to pathological microbial dislocation [217]. This is caused (in part) by the depletion of ILC3s in response to HIV infection, which is dependent on plasmacytoid DCs, IFN-I, and FASL (Fig. 4C) [218]. ILC3 depletion has also been observed in SIV-infected macaques [219–221]. SIV infection also induces mucosal damage, consistent with the key role that ILC3s play in maintaining barrier function [219]. Administration of combination antiretroviral therapy (cART) reversed ILC3 depletion in humanized mice [218]. However, HIV patients receiving cART have decreased levels of ILC1s and ILC3s in the ileum and colon, which are associated with increased serum markers of intestinal damage, suggesting that sustained ILC depletion does not require viral replication [222]. A subset of ILC1s expresses CD4 and can be a direct target of HIV, indicating that direct infection with ILC1s may contribute to decreased numbers of these cells [223]. Postnatal transmission of SIV in rhesus macaques is associated with decreased gut ILC3s and increased oral mucosa ILC3s, indicating tissue-specific depletion of ILC3s [224]. However, in both the oral and gut mucosa, ILC3s had decreased IL-22 production but increased degranulation and type I cytokine production, indicating SIV-induced ILC plasticity. However, it is unclear whether the HIV-induced loss of barrier integrity results from virus-mediated subversion or off-target immunopathology. Nevertheless, HIV infection highlights the role that ILC3s play in maintaining gut homeostasis and their role in preventing microbial dislocation under conditions in which CD4 + T-cell immunity is impaired.

## Genital herpes infection

Genital HSV-2 infections cause recurrent genital ulcers and inflammation and increase the risk of acquiring HIV [225]. CCR5-dependent NK cell recruitment is required for viral control in a mouse model of vaginal HSV2 infection [226]. Moreover, it was shown that HSV-2 causes neutrophils to produce IL-18 in an IFN-I-dependent manner, which causes NK cell-mediated epithelial ulceration in genital tissue [227, 228]. In this model, IL-18 induces murine NK cells to secrete granzyme B, which triggers epithelial ulceration [228]. HSV-2 causes greater genital pathology than HSV-1, which is in part due to an early burst of IFNγ produced by NK cells that drives a protective T-cell response [229]. Taken together, these studies illustrate the protective and pathogenic role that NK cells play in the immune response to genital herpes infection.

## Infection of the central nervous system

The central nervous system (CNS) and the brain in particular have long been considered immune-privileged sites, but the identification of bone marrow-derived immune cells in specialized niches within the CNS has revised this view [230]. These immune cells include all ILC subsets and they have been implicated in CNS infections, including herpesvirus, flavivirus, coronavirus, and alphavirus infections [231].

HCMV can cause congenital viral infections, which have been associated with CNS disabilities [232]. Congenital infection with MCMV causes encephalitis and altered cerebellar development, which is associated with CXCL9/CXCL10-dependent influx of type I ILCs [233, 234]. While NK cells contribute to viral control in adult mice, in newborn mice, NK cells are unable to mediate viral control [235]. Instead, type I ILCs cause immunopathology in response to congenital MCMV infection [234]. Consistent with these findings, infiltration of NKp46+ cells correlates with the severity of lesions in HCMV-infected fetal brains [236]. In addition to herpesvirus infection, a role for type I ILCs in inducing immunopathology in the brain has also been observed for the alphavirus Semliki Forest virus [237]. However, in the context of coronavirus mouse hepatitis virus and flavivirus Zika virus infection, infiltration of NK cells in the brain has been linked to viral control [238, 239]. Thus, type I ILCs in the CNS can mediate immunopathology and/or viral control depending on the type of virus.

ILC2s have been reported to induce T-cell-dependent CNS demyelination in response to infection with a herpes simplex virus (HSV) modified to constitutively express IL2 [240]; however, these observations were based on a genetically modified virus, and the physiological relevance of these results remains to be determined. These findings implicate ILC2s in virus-induced immunopathology within the CNS.

Neuroadapted Sinbis virus (NSV) is an alphavirus that can cause encephalomyelitis, which is exacerbated in the absence of IL-10 [241]. ILC3s in the brain produce increased TGFβ production, which is associated with increased IL-17 production [242], indicating that ILC3s contribute to immunopathology in the CNS in response to NSV. Taken together, all ILC subtypes have been implicated in virus-induced immunopathology of the CNS, highlighting the delicate nature of this organ.

## Final conclusions

There is a substantial body of evidence showing that the different ILC subtypes play critical and specialized roles in the innate immune response to virus infection. This review highlighted the role that different ILC subsets play in virus infection within tissues. However, ILCs have been implicated in other viral infections, such as infections with papilloma and Epstein–Barr viruses, but the related tissue-specific immunity is largely unexplored. As we are starting to appreciate the tissue-specific role that ILCs play in antiviral immunity, the number of viruses that involve specific ILC subsets is likely to increase.

The antiviral ILC response to acute virus infection starts within 12-48 hours after infection, significantly before the adaptive immune response initiates an antigen-specific response [243]. ILC1s act during the earliest phases of this response, as illustrated by ILC1 IFNγ production in response to MCMV infection, which is detectable as early as 12 hours post infection [116]. This study also revealed that lung ILC1s also produce IFNγ in response to intranasal infection with other viruses, including Sendai and influenza virus, indicating that the antiviral functions of tissue-resident ILC1s are preserved across tissues. NK cells can also respond very early to virus infection, and NK cell IFNγ in the spleen has been reported to respond as early as 12 hours post infection, peaking at approximately 36 hours in the spleen [71, 244–246]. Thus, both NK cells and ILC1s respond to virus infection at the earliest phases of infection. ILC1s have been suggested to mediate their antiviral effects before NK cells start eliminating virus-infected cells [116], which is an intriguing hypothesis because tissue-resident ILC1s are ready at the site of infection, while NK cells can circulate toward the site of infection to mediate their full antiviral functions. Further studies investigating both antiviral NK and ILC1 immunity using genetic animal models with specific NK cell versus ILC1 deficiencies are needed to further characterize these very early responses to virus infection.

As reviewed here, ILCs also play a crucial role after virus infection is resolved and during chronic phases of infection. In particular, interest in these processes in ILC2s has increased [31]. The type II cytokines produced by ILC2s can play a role in modulating the immune response, including the suppression of type I immunity and the production of type II cytokines such as IL-5, which mediate eosinophil maintenance [11, 124]. Moreover, ILC2s can produce AREG, which can directly impact tissue repair [127, 247]. AREG impacts tissue repair through diverse mechanisms, including macrophage and tissue differentiation (reviewed in [248, 249]). As reviewed here, ILC2s contribute to tissue repair in response to influenza infection in the lung and LCMV infection in the liver, indicating that ILC2s can contribute to tissue repair across tissues against multiple viruses. As ILC2s are instructed by cytokines, such as IL-33 and TLSP, it is likely that ILC2s can mediate tissue repair in response to a wide range of viruses. However, on the flip side, ILC2s, including influenza, RSV, and rhinoviruses, are implicated in virus-induced asthma. Thus, ILC2s mediate both protective and pathological immunity, even against the same virus. Additional research is needed to further unravel the protective versus pathological functions of ILC2s and identify potential therapeutic targets.

Data showing that ILC3s can impact virus infection are emerging. ILC3s mediate protective immunity against rotavirus infections (in part) by inducing interferon-stimulated genes. As ILC3s also impact the gut microbiota, modulation of the microbiota by ILC3s might also impact disease progression, which requires further study. Moreover, HIV has been reported to reduce the number of ILC3s in the intestine, which is associated with decreased barrier function. These data suggest that ILC3s are involved in maintaining barrier function during virus infection. Depletion of ILC3s in response to virus infection likely extends to other viruses as well. For example, HCMV infection reportedly impaired the generation of ILC3s in an in vitro model of ILC differentiation [250]. Thus, ILC3s can mediate antiviral immunity through IL-22 production and are key in maintaining barrier function to prevent systemic infections.

Taken together, even though the different members of the ILC family contribute to antiviral immunity, the individual members employ unique effector functions to impact viral immunity within the tissue microenvironment.
